# Intralesional rose bengal in melanoma elicits tumor immunity *via* activation of dendritic cells by the release of high mobility group box 1

**DOI:** 10.18632/oncotarget.9247

**Published:** 2016-05-09

**Authors:** Hao Liu, Pasquale Patrick Innamarato, Krithika Kodumudi, Amy Weber, Satoshi Nemoto, John L. Robinson, Georgina Crago, Timothy McCardle, Erica Royster, Amod A. Sarnaik, Shari Pilon-Thomas

**Affiliations:** ^1^ Department of Immunology, H. Lee Moffitt Cancer Center and Research Institute, Tampa, Florida, USA; ^2^ Flow Cytometry Core Facility, H. Lee Moffitt Cancer Center and Research Institute, Tampa, Florida, USA; ^3^ Department of Translational Science, H. Lee Moffitt Cancer Center and Research Institute, Tampa, Florida, USA; ^4^ Department of Cutaneous Oncology, H. Lee Moffitt Cancer Center and Research Institute, Tampa, Florida, USA; ^5^ Department of Pathology, H. Lee Moffitt Cancer Center and Research Institute, Tampa, Florida, USA; ^6^ Department of Cutaneous Data Management, H. Lee Moffitt Cancer Center and Research Institute, Tampa, Florida, USA

**Keywords:** rose bengal, high mobility group box 1, dendritic cells, melanoma, intralesional therapy

## Abstract

Intralesional (IL) therapy is under investigation to treat dermal and subcutaneous metastatic cancer. Rose bengal (RB) is a staining agent that was originally used by ophthalmologists and in liver function studies. IL injection of RB has been shown to induce regression of injected and uninjected tumors in murine models and clinical trials. In this study, we have shown a mechanism of tumor-specific immune response induced by IL RB. In melanoma-bearing mice, IL RB induced regression of injected tumor and inhibited the growth of bystander lesions mediated by CD8^+^ T cells. IL RB resulted in necrosis of tumor cells and the release of High Mobility Group Box 1 (HMGB1), with increased dendritic cell (DC) infiltration into draining lymph nodes and the activation of tumor-specific T cells. Treatment of DC with tumor supernatants increased the ability of DCs to stimulate T cell proliferation, and blockade of HMGB1 in the supernatants suppressed DC activity. Additionally, increased HMGB1 levels were measured in the sera of melanoma patients treated with IL RB. These results support the role of IL RB to activate dendritic cells at the site of tumor necrosis for the induction of a systemic anti-tumor immune response.

## INTRODUCTION

Immunotherapeutic strategies incorporating intralesional (IL) therapy to elicit tumor-specific immune responses constitute a viable option for cutaneous neoplasms. These strategies can induce both local and systemic tumor regression. Intratumoral injection of dendritic cells (DCs), IL-2, GM-CSF, or Bacille Calmette-Guérin (BCG) have been shown to enhance anti-tumor immunity in both melanoma-bearing mice and in patients with advanced melanoma [1–6].

Rose bengal (RB) was originally used as a staining agent by ophthalmologists and in liver function studies [7, 8]. RB has a direct cytotoxic effect on microorganisms and cancer cells [9–15]. In tumor cells, RB selectively passes through the cell membrane and accumulates in the lysosomes resulting in autolysis [10]. IL injection of RB (also referred to as PV-10, an investigational drug formulation of 10% RB in normal saline) into tumors has been shown to elicit regression of both treated and untreated tumors [12, 16]. In murine models, IL PV-10 induced T cell mediated tumor-specific immune responses in MT901 breast cancer and in B16 melanoma [16]. In a phase I study in metastatic melanoma patients, IL PV-10 was well tolerated and led to a 48% objective response (OR) in treated lesions and a 27% OR in untreated lesions [12]. A recent phase II clinical trial of PV-10 demonstrated a similar OR in treated and untreated lesions [17]. Additional evidence of systemic response was supported by the regression of untreated visceral lesions in a few patients. However, the underlying mechanism of induction of tumor immunity remains unknown.

Dying cancer cells release soluble molecules known as Damage-Associated Molecular Pattern Molecules (DAMPs), which are recognized by pattern recognition receptors (PRRs) [18]. DAMPs serve as powerful immunological adjuvants for cancer therapy. DAMPs can promote phagocytosis, antigen-presentation, and inflammasome activation in DCs, collectively fostering T cell priming against antigens [19]. DAMPs include members of the Heat Shock Protein (HSP) family, the S100 proteins, ATP, IL-1α, and High Mobility Group Box 1 (HMGB1, amphoterin) [20–25]. As the best characterized DAMP, HMGB1 is a ubiquitous protein bound to DNA in almost all eukaryotic cells. It can be membrane-bound or secreted into the extracellular space as a cytokine-like factor, or can be passively released by necrotic, apoptotic and autophagic cells [26–29]. HMGB1 release by dying tumor cells can lead to the activation of DCs and prevention of tumor progression [25, 30–32]. HMGB1 putative receptors include the Receptor for Advanced Glycation End-products (RAGE), Toll-like Receptor 2 (TLR2), TLR4 and T cell immunoglobulin and mucin protein 3 (TIM-3) [33–35].

In this study, we investigated whether IL PV-10 leads to the release of DAMPs contributing to the induction of an anti-tumor immune response.

## RESULTS

### IL PV-10 elicits a tumor-specific immune response

**Figure 1:**
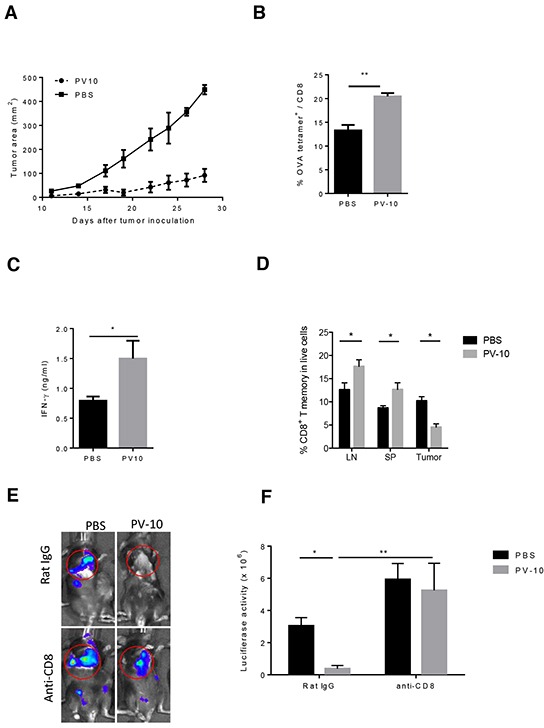
IL injection of PV-10 elicits tumor-specific immunity in melanoma-bearing mice M05 cells (3e5) were injected into one flank of C57BL/6 mice on day 0. PV-10 or PBS (50 μl) was injected IL on day 7 (n=4 mice / group). **A.** Tumor growth. **B.** The percentage of CD8^+^, OVA tetramer^+^ T cells was measured in the DLNs after 8 days by flow cytometry. Data are representative from two independent experiments and are shown the mean number ± SEM. **C.** Mice were re-challenged with 3e5 M05 cells s.c. on the opposite flank on day 7 and 50 μl PV-10 or PBS were injected IL into the initial tumor lesion on days 7 and 17 (n=4). On day 23, splenocytes were expanded with 20 ng/ml IL-15 and IL-21 and 1 μg/ml SIINFEKL for 7 days and then co-cultured with M05 cells. IFN-γ production was measured after 48 hours. Data are presented as mean ± SEM from three independent experiments. **D.** Mice were inoculated with 3e5 M05 cells on both flanks (n=4 mice/group). On day 7, PV-10 or PBS were injected IL into the left flanks, and 10 days later, CD8^+^ T memory cells (CD44^hi^ CD62L^hi^ and CD44^hi^ CD62L^lo^ cells) were measured in LNs, spleens and right flank tumor. Data are the representative from two independent experiments and presented as mean ± SEM. **E–F.** Mice were injected with 1e5 B16 cells s.c. and 4e5 luciferase-tagged B16-F10-luc cells *i.v*. (n=6-9 mice / group). On day 7, 50 μl PV-10 or PBS were injected IL. Mice were treated with 100 μg of purified CD8 depleting antibody (2.43) or control rat IgG antibody 2 and 3 days prior to PV-10 treatment and twice per week until day 21. E. Representative Xenogen imaging results are shown. F. Data are the representative from two independent experiments and are shown the mean number ± SEM of luciferase intensity in the lung region. * *p*< 0.05, statistically significant versus control; ** *p*<0.01. *P* values were determined by an unpaired student *t*-test.

To investigate the underlying mechanism of the tumor-specific immune response elicited by PV-10, C57BL/6 mice were injected subcutaneously (s.c.) with M05 tumor cells expressing the ovalbumin (OVA) protein [36]. Similar to our previous findings in the B16 model, IL injection of PV-10 directly inhibited tumor growth (Figure 1A) [16]. Moreover, IL PV-10 led to increased OVA-specific CD8^+^ T cells in the draining lymph nodes (DLNs) of PV-10-treated mice, compared to the PBS-treated group (Figure 1B). To determine whether IL PV-10 induced T cells with memory characteristics, splenocytes from mice treated twice with IL PV-10 were cultured *in vitro* in the presence of OVA peptide and media supplemented with the cytokines IL-15 and IL-21, which are required for maintaining CD8^+^ T memory cells [37]. T cells from PV-10-treated mice demonstrated a ca. 2 fold increase in secretion of IFN-γ in response to M05 cells, compared to T cells isolated from PBS-treated mice (Figure 1C). To further confirm the induction of memory T cells, spleens, lymph nodes (LNs), and tumors were collected from mice 10 days after IL PBS or PV-10 injection. Memory T cells (CD44^hi^ CD62L^hi^ and CD44^hi^ CD62^low^) were increased in the LNs and spleens of mice treated with PV-10 compared to mice treated with PBS (Figure 1D). In contrast, there were decreased T memory cells in bystander tumors of treated mice. These results suggest that IL PV-10 can induce tumor-specific T cells with memory characteristics in M05 melanoma-bearing mice.

We next investigated whether CD8^+^ T cells mediate the tumor-specific immune response elicited by IL injection of PV-10. Using a lung metastasis model described previously, mice received B16F10 cells s.c. on day 0 to establish a solitary tumor on the flank and B16-F10-luc cells intravenously (i.v.) to establish multiple lung lesions [16]. On day 7, the palpable flank tumors were treated IL with 50 μl PBS or PV-10. Tumors in the lungs were monitored by bioluminescent imaging on day 21 (Figure 1E). All mice that received IL PBS displayed growth of tumor in the flank and multiple lung lesions. In contrast, mice that received IL PV-10 developed fewer lung lesions (Figure 1F, p<0.05 compared to PBS-treated mice). To examine the role of CD8^+^ T cells, mice received a CD8 depleting antibody (clone 2.43) or the isotype antibody (Rat IgG). After depletion of CD8^+^ T cells, mice receiving IL PV-10 developed equivalent numbers of lung lesions compared to mice receiving PBS treatment (Figure 1F). These results show that CD8^+^ T cells are crucial for the tumor-specific immune response induced by IL injection of PV-10.

**Figure 2:**
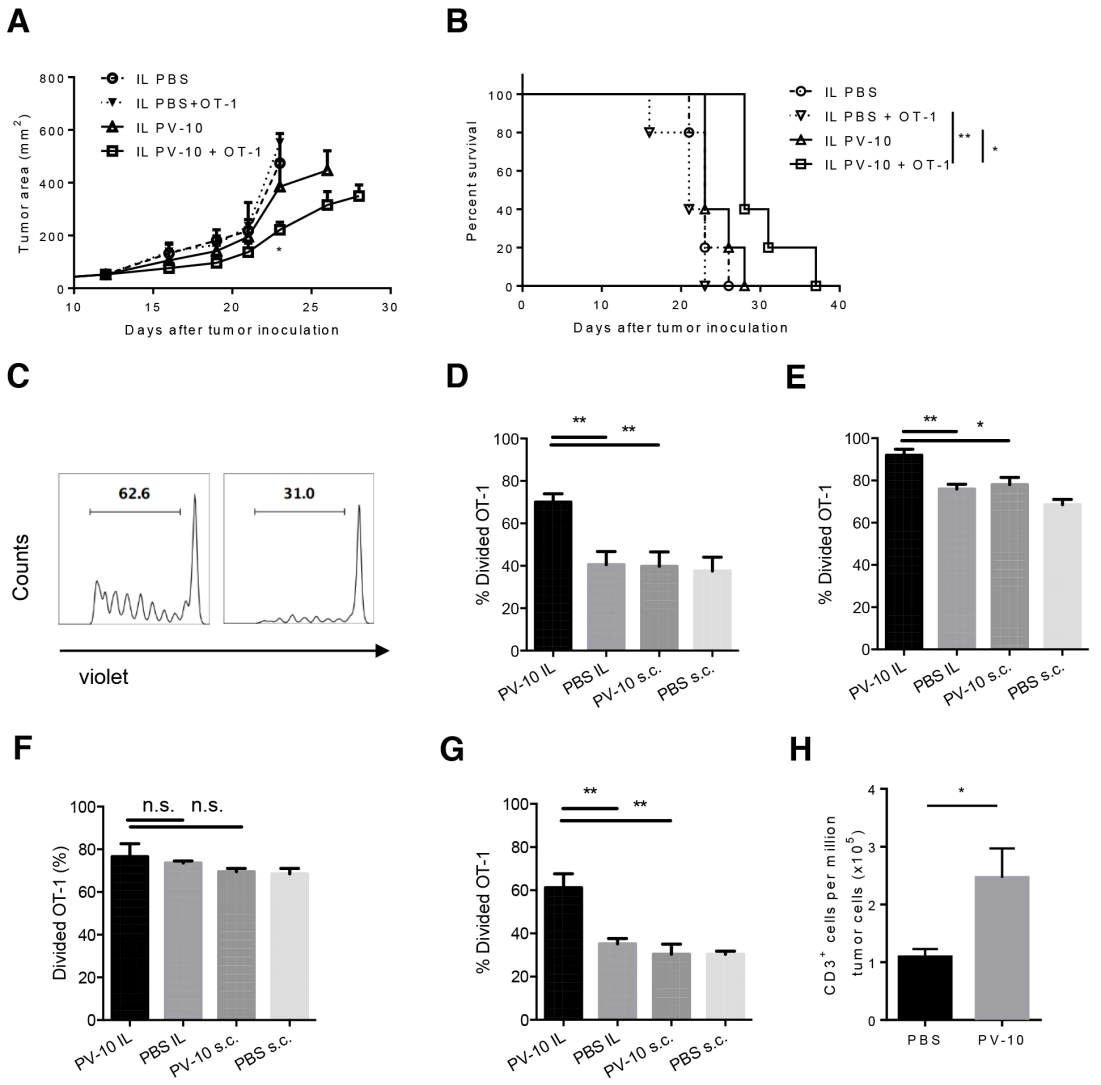
IL injection of PV-10 facilitates the proliferation of tumor-specific CD8+ T cells in M05-bearing mice **A–G.** M05 cells (3e5) were injected into one flank of C57BL/6 mice on day 0. On day 13, 50 μl PV-10 or PBS were injected IL or s.c. in the opposite flank without tumor. Four hours later, 2e6 violet labeled CD45.1^+^ OT-1 T cells were i.v. injected. Tumor growth (A) and survival (B) of mice were monitored (n=5 mice / group). After 4 days, cells from spleens (C, D), tumors (E), draining LNs (F) and non-draining LNs (G) were stained with CD45.1 and CD45.2 antibodies. Representative histograms of violet dye dilution show the progenies of CD45.1^+^ T cells, which have at least one division, after discrimination of dead cells (C). Data are presented as mean ± SEM from three independent experiments (n=5 mice / group). **H.** 3e5 M05 cells were injected s.c. into both flanks of C57BL/6 mice on day 0 (n=7). On day 7, 50 μl of PV-10 or PBS were injected IL in the left flank and OT-1 cells were transferred into mice. After an additional 4 days, CD3^+^ cells were measured in the bystander (right flank) tumor. Data are presented as mean ± SEM from two independent experiments. *P* values were determined by an unpaired student *t*-test (A, D-H) or a log-rank test (B). *p< 0.05 versus control; **p<0.01.

To monitor CD8^+^ T cell responses *in vivo*, PV-10 or PBS was IL injected into s.c. M05 tumors on day 13, followed by injection of violet-dye labeled OT-1 cells. The average tumor size was 50 mm^2^ on day 13 yielding sufficient tumor material to dissect for examination of T cell proliferation after IL PV-10. OT-1 T cells are CD8^+^ T cells that specifically recognize the SIINFEKL peptide derived from the OVA protein. Adoptive transfer of OT-1 cells alone or treatment with IL PV-10 alone was not sufficient to prevent tumor growth when treatment began at day 13. The combination of IL injection of PV-10 and transfer of OT-1 cells significantly reduced tumor progression and increased survival (Figure 2A–2B). We next measured the proliferation of OT-1 T cells in tumors, LNs, and spleens after IL PV-10 injection. In the spleens of PV-10 treated mice, on day 4 after transfer, more than 60% of OT-1 T cells demonstrated at least one division, hereafter called “divided” T cells (Figure 2C, left panel). In contrast, there were fewer divided T cells in the spleens of PBS-treated mice (Figure 2C, right panel). IL PV-10-treated mice exhibited increased proliferation of OT-1 T cells in the spleen compared to mice treated with IL PBS (Figure 2D). However, when PV-10 was injected s.c. into the opposite flank where there was no tumor, proliferation of OT-1 T cells was equivalent to proliferation of OT-1 T cells in mice that received PBS in either the opposite flank or IL into tumor. At the tumor site, OT-1 cells robustly proliferated in PV-10- or PBS-treated mice (Figure 2E). This may be due to the relatively high OVA protein expression by the M05 tumor. A robust OT-1 T cell proliferation was also observed in the tumor draining LNs and no difference was seen between PBS- and PV-10-treated mice (Figure 2F). Differences in OT-1 T cell proliferation were measured in the non-draining LNs of mice treated with IL PV-10 compared to mice treated with IL PBS or treated on the opposite flank (Figure 2G). These data suggest that IL PV-10 can enhance T cell proliferation.

Next, we examined whether IL PV-10 increased the infiltration of T cells into tumors. As IL PV-10 can ablate injected tumor, a bilateral tumor model was used to monitor the T cell infiltration. Bilateral M05 tumors were established in C57BL/6 mice. After 7 days, mice received IL PV-10 in the left flank tumor, and four hours later were i.v. injected with OT-1 cells. Four days later, bystander tumors were collected and T cell infiltration was measured. As shown in Figure 2H, there was a significant increase in T cells infiltrating the bystander tumor. Together, these data support that IL injection of PV-10 can boost T cell infiltration in tumors.

### IL PV-10 leads to DC activation

**Figure 3:**
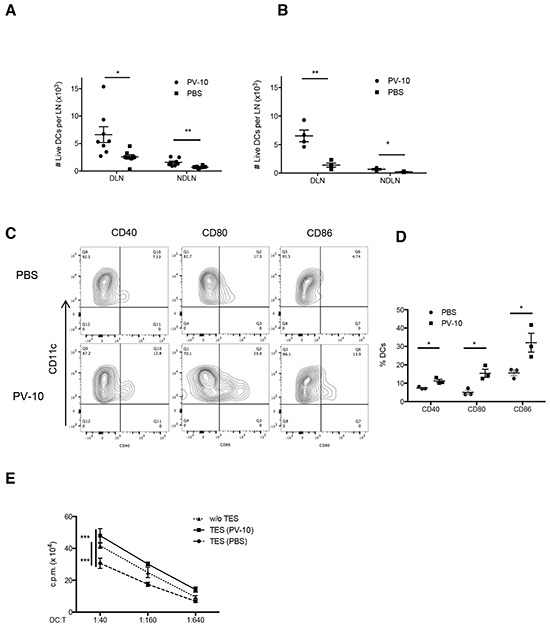
Increased DC infiltration into LNs after IL PV-10 **A–D.** M05 cells (3e5) were injected into C57BL/6 mice. PV-10 or PBS (50 μl) was injected IL on day 7. A. The number of DCs (CD11c^+^ MHC II^+^) from tumor draining LNs or NDLNs was measured by flow cytometry after 18 hours (n=6 mice / group). B. FITC-OVA was injected intratumorally 4 hours after IL injection. The cell number of FITC^+^ DCs from DLNs or NDLNs was measured by flow cytometry after 18 hours. Data are presented as mean ± SEM from three independent experiments (n=4 mice / group). C-D. DCs from pooled DLNs were enriched after 18 hours and the expression of CD40, CD80, and CD86 were examined on CD11c^+^ MHC II^+^ cells. The representative FACS plots (C) and summarized data (D) from three independent experiments are shown. Data represent the mean ± SEM. **E.** BM-derived DCs were incubated for 2 days with complete media (w/o TES) or 20% tumor explant supernatants (TES) from M05 melanoma-bearing mice treated with IL PV-10 or PBS, then pulsed with OVA protein and co-cultured with OT-1 T cells at different ratios for 3 days. OT-1 proliferation was examined by the [3-H]-thymidine incorporation in the last 16 hours. Data are shown as mean ± SEM of five replicates for each of two independent experiments. *P* values were determined by an unpaired student *t*-test (A, B, D, E). *p< 0.05 versus control; **p<0.01; ***p<0.001.

Because DCs are capable of priming T cells, we next examined DCs in the spleens and LNs of mice treated with IL PV-10. At 24 hours after IL PV-10 injection, the number of infiltrating DCs increased in both draining LNs (DLNs) and non-draining LNs (NDLNs) (Figure 3A). The overall total number of cells in the LNs was not significantly changed after treatment. After 72 hours, the number of infiltrating DCs in DLN of PV-10-treated mice decreased to the baseline level and was equivalent to the level in the PBS-treated mice (data not shown), suggesting that the infiltration of DCs is transient. To examine whether DCs migrated from the site of tumor, OVA protein labeled with FITC (FITC-OVA) was directly injected into tumor 4 hours after IL injection of PV-10 or PBS. After 18 hours, lymph nodes were collected. We measured increased FITC^+^ DCs in the DLNs but not in the NDLNs of the PV-10-treated mice (Figure 3B). To assess DC activation, DLNs were collected from M05 bearing mice treated with IL PV-10 or PBS. DCs were enriched and stained with co-stimulatory markers. DCs isolated from the DLNs of IL PV-10 treated mice expressed higher levels of activation markers, including CD40, CD86 and CD80, compared to PBS-treated mice (Figure 3C–3D). Together, these studies support a role for IL PV-10 to induce DCs to take up antigens at the tumor site, infiltrate into the DLN, and become functionally mature.

We next tested whether IL injection of PV-10 led to DC activation. Bone marrow cell (BM)-derived DCs were co-cultured with supernatants of M05 tumors that were previously treated with IL PV-10 or PBS. DCs were then pulsed with OVA protein and co-cultured with OT-1 T cells. DCs cultured with supernatants derived from M05 tumors isolated from PV-10-treated mice induced increased proliferation of OT-1 T cells, compared to DCs cultured with supernatants derived from M05 tumors from PBS-treated mice (Figure 3E). These results suggest that PV-10-treated tumors may release factors that activate DCs.

### PV-10 treatment increases DC activation via HMGB1

**Figure 4:**
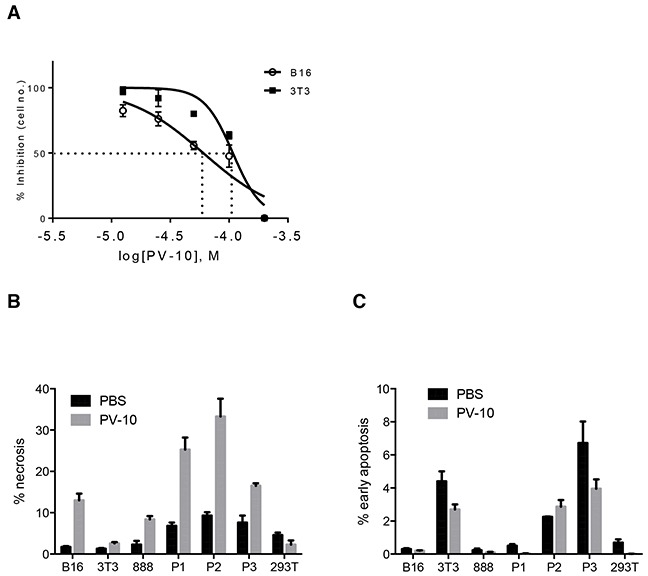
PV-10 leads to tumor cell death through necrosis **A.** Mouse melanoma B16 cells and mouse 3T3 fibroblasts were cultured with various doses of PV-10 for 48 hours to determine the IC_50_. Data are shown as mean ± SEM of triplicates. B16 cells, 3T3 fibroblasts, human 888 melanoma cells, three human primary melanoma cells (P1, P2, P3) and human embryonic kidney 293T cells, were treated with 50 μM PV-10 for 48 hours. **B.** Necrosis (DAPI^+^) and **C.** early apoptosis (Annexin V^+^ DAPI ^−^) was measured by flow cytometry. Data are presented as mean ± SEM of triplicates from two independent experiments.

To examine whether tumor death induced by IL PV-10 is linked to the activation of DCs, we first investigated how PV-10 induces cell death. As shown in Figure 4A, PV-10 resulted in a dose-dependent cytotoxicity in B16 melanoma cells, with an IC50 value of 60 μM after 48 hours of treatment. There was less cytotoxicity in mouse NIH3T3 fibroblasts, with an IC50 value of 110 μM after 48 hours of treatment with PV-10 (Figure 4A). The IC50 value of PV-10 on B16 and 3T3 cells was similar at 6, 12, and 24 hours (data not shown). There was a significant increase in necrosis (DAPI^+^) of B16 cells, human 888 melanoma cells and human primary (P1-3) melanoma cells after 48 hours of treatment with 50 μM of PV-10 (Figure 4B). However, after 48 hours, a relatively small proportion of cells were in early apoptosis (Annexin V^+^ DAPI^−^, Figure 4C), with no difference after 6, 12, and 24 hours of treatment (data not shown). This indicates that treatment with 50 μM of PV-10 leads to tumor cell death through necrosis rather than apoptosis. Little necrosis or apoptosis was measured in 3T3 fibroblasts or human embryonic kidney 293T cells in the presence of the same dose of PV-10 (Figure 4B and 4C). These studies suggest that PV-10 can kill tumor cells at a dose that is not toxic to non-tumor cells.

**Figure 5:**
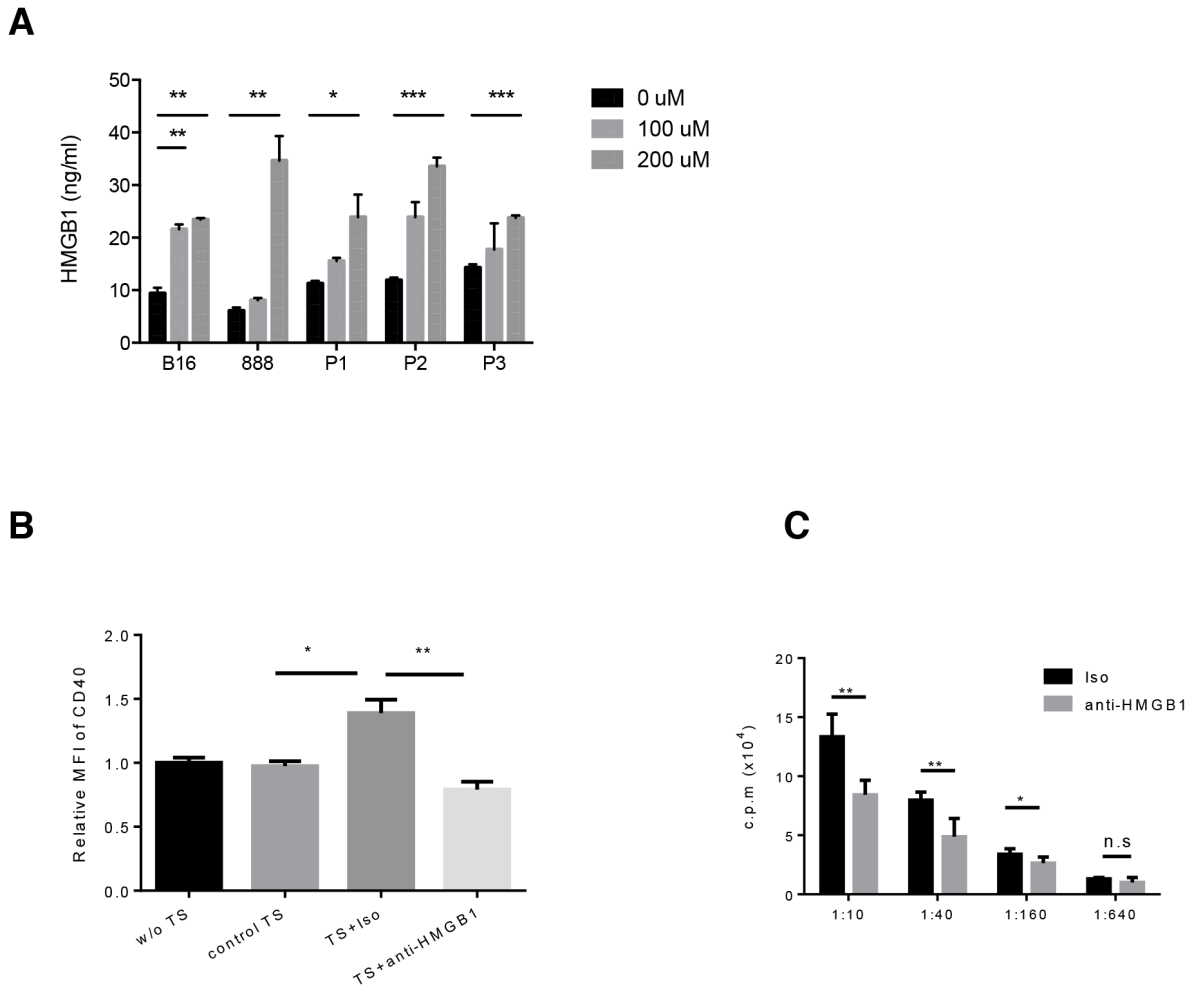
PV-10 treatment leads to the release of HMGB1 from melanoma cells Murine B16, human 888 melanoma cells and three human primary melanoma cells (P1, P2, P3) were treated with various doses of PV-10 for 48 hours. **A.** HMGB1 in cell supernatants was detected by ELISA. Data are shown as mean ± SEM of triplicates. **B.** BM-derived DCs were incubated with complete media (w/o TS), or B16 cell supernatants supplemented with PV-10 (control TS), or supernatants of B16 cells which were pre-incubated with 100 uM PV-10 in the presence of HMGB1 neutralizing antibody (TS + anti-HMGB1) or isotype control (TS + Iso) for 2 days. Cells were stained with antibodies against CD40 and CD11c and analyzed by flow cytometry. The mean fluorescence intensity (MFI) of CD40 of DCs is shown. Data are shown as mean ± SEM of triplicates and are representative for three independent experiments. **C.** BM-derived DCs were incubated with TES from M05 melanoma-bearing mice treated with IL PV-10 in the presence of HMGB1 neutralizing antibody or isotype control for 2 days, pulsed with OVA protein and co-cultured with OT-1 T cells at different ratios for 3 days. OT-1 proliferation was examined by [3-H]-thymidine incorporation during the final 16 hours of culture. Data are shown as mean ± SEM of five replicates in each of two independent experiments. *P* values were determined by an unpaired student *t*-test. *p< 0.05 versus control; **p<0.01, ***p<0.001, n.s. not significant.

We next examined the factors that are released by tumor cells after treatment with PV-10. It has been shown that necrosis is associated with the disruption of the integrity of the cell membrane and the uncontrolled release of cytosolic contents into extracellular space, including DAMPs such as HMGB1, IL-1a, and HSP proteins. Murine B16 melanoma, human 888 melanoma cells and human primary melanoma cells were treated with 0, 100 or 200 μM PV-10 for 48 hours. The release of DAMPs including HMGB1, HSP70, HSP90 and IL-1a were measured by ELISA or western blot. HSP70 and IL-1a were not detected and HSP90 was unchanged after treatment with PV-10 (data not shown). HMGB1 was measured in the supernatants of B16, primary melanomas, and 888 cells in a dose-dependent manner (Figure 5A).

To determine if secreted HMGB1 contributed to DC activation, BM-derived DCs were incubated for 2 days with 20% supernatant from B16 cells treated with PV-10 in the presence of HMGB1 neutralizing antibody or isotype control antibody. Tumor supernatant from PV-10-treated cells led to DC maturation, with the up-regulation of surface CD40. Neutralization of HMGB1 in supernatants significantly decreased CD40 expression (Figure 5B). Treatment of DCs with PV-10 directly did not change CD40 expression, suggesting that PV-10 itself does not lead to DC maturation. Other co-stimulatory markers on DCs, including CD86 and CD80, were unchanged (data not shown).

To compare the antigen presentation capacity of DCs, BM-derived DCs were incubated with the supernatant from M05 tumor treated with IL PV-10 in the presence of HMGB1 neutralizing antibody or isotype antibody for 2 days. The pre-treated DCs were pulsed with OVA protein and co-cultured with OT-1 T cells. T cell proliferation was measured. The blockade of HMGB1 decreased the ability of DCs to stimulate OT-1 T cell proliferation (Figure 5C). This suggests that treatment of melanoma cells with PV-10 leads to the release of HMGB1, and the activation of DCs.

### IL PV-10 leads to HMGB1 increase in the sera of melanoma patients

**Figure 6:**
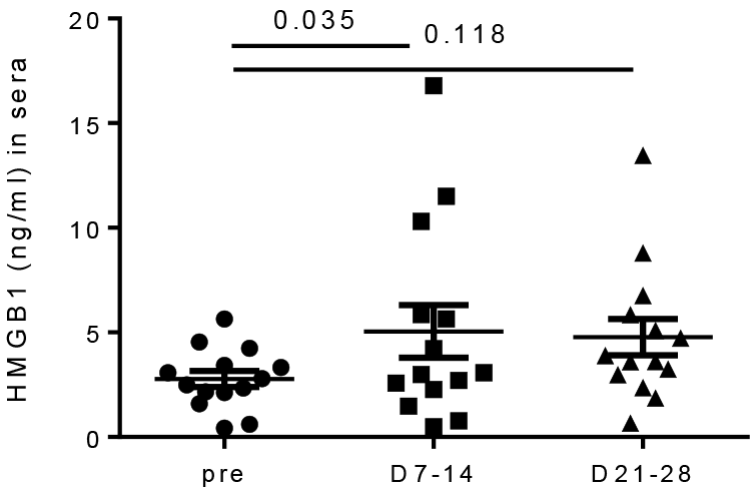
Increased HMGB1 levels in sera of melanoma patients after IL PV-10 Sera was collected from melanoma patients before and one week after IL injection of PV-10. Data are shown as mean ± SEM (n=14 patients). *P* values were determined by a Wilcoxon matched-pairs signed rank test.

To determine whether HMGB1 release is relevant in melanoma patients treated with IL PV-10, we compared the level of HMGB1 in the serum of patients obtained before and after treatment with IL PV-10 (see “Materials and Methods” and Supplementary Figure S1). IL PV-10 led to tumor regression in both an injected and an uninjected bystander lesion, shown by IHC staining for the melanoma antigen Melan-A/MART-1 (melA) in the biopsy specimens (Supplementary Figure S1B-S1C). Notably, the concentration of HMGB1 was significantly increased in serum collected 7-14 days after treatment with IL PV-10 (Figure 6). Therefore, HMGB1 may contribute to the bystander effect induced by IL PV-10 in patients with metastatic melanoma.

The percentage of infiltrating immune cells in PV-10 treated and bystander lesions were compared before and after treatment with IL PV-10. However, very few infiltrates were detected in the lesions that completely regressed, and no significant changes were measured. Thus an alternative method was used to compare the presence of immune subsets in peripheral blood mononuclear cells (PBMCs) before and after treatment. There was a statistically significant increase in circulating CD8^+^ T cells, CD4^+^ T cells, and NKT cells after PV-10 treatment (Supplementary Figure S2). There was no difference in circulating NK cells, MDSC, CD4^+^FOXP3^+^ regulatory T cells or plasmacytoid DCs before and after treatment (data not shown).

To determine whether we could measure tumor-specific T cell responses after PV-10 treatment, CD8^+^ T cells were purified from PBMC collected from 7 patients before and after treatment. T cells were co-cultured with autologous or HLA-matched melanoma cell lines *in vitro* for 24 hours and supernatants were collected. A significant increase in IFN-γ production was measured in the CD8^+^ T cells isolated after treatment with IL PV-10 in 5 patients out of 7 patients that were tested. No change was measured when CD8^+^ T cells were co-cultured with HLA-mismatched cell lines (Supplementary Figure S3). These preliminary results support the role of IL PV-10 treatment to induce a systemic anti-tumor immune response in patients with metastatic melanoma.

## DISCUSSION

Melanoma incidence rates have increased rapidly in the United States over the past 30 years and is the fifth most common cancer in men and the seventh most common cancer in women [38]. IL therapy is a promising treatment modality for patients with dermal and/or subcutaneous metastatic melanoma. Importantly, it may induce not only local tumor regression but also a systemic anti-tumor immune response. In a recent clinical trial in metastatic melanoma patients, IL PV-10 led to a 50% objective response rate with mild to moderate side effects [17]. In treated patients, 8% had no evidence of disease after 52 weeks and 26% experienced complete regression in bystander lesions. However, the mechanism by which IL PV-10 leads to systemic anti-tumor immunity is unknown.

In this study, we showed that IL PV-10 led to the necrosis of melanoma cells and the release of HMGB1. These data are consistent with the observation that HMGB1 was passively released from photosensitized HeLa cells treated with a rose bengal analog [39]. Pretreatment with rose bengal acetate led to apoptosis and autophagy and the secretion of HSP70, HSP90 and HMGB1. In contrast, our results showed that PV-10 treatment induced necrosis in melanoma cells and the secretion of HMGB1, but not HSP70, while the amount of HSP90 was unchanged. This discrepancy may be explained by differences in response to RB and its acetate analog, dose of test article, differences in the cell lines used, or mechanisms of ablative and photodynamic therapies. Moreover, HMGB1 levels in the sera of patients were increased after IL PV-10. This is in line with another study that showed increased HMGB1 levels in the serum of cancer patients after chemoradiation; notably, HMGB1 levels were increased in patients with antigen-specific T cell responses and higher expression of HMGB1 in resected tumor samples was correlated with better survival [40].

Maturation of DCs is crucial for priming CD8^+^ T cells [41]. HMGB1 has been shown to be important for activation of myeloid and plasmacytoid DCs [25, 31, 42–46]. In our model, DC maturation with up-regulation of CD40, CD80 and CD86 was measured in tumor draining LN after IL PV-10. Furthermore, our study showed that HMGB1 in the supernatant of tumor cells treated with PV-10 was responsible for the up-regulation of CD40 expression on BM-derived DCs and for the increased ability of DC to stimulate T cell activation. It has been shown that short-term CD40 signaling augments DC migration to tumor-draining LNs and induced protective immunity. Moreover, HMGB1 has been shown to enhance DC responses to CCL9 and CXCL12 [47]. Interactions between HMGB1 and RAGE can induce the migration of s.c. injected DCs into DLNs [48]. In our study, IL PV-10 increased the number of DCs migrating from the tumor site into the draining LNs.

In this study, we have shown a mechanism of tumor-specific immune response induced by IL PV-10. In melanoma-bearing mice, IL PV-10 induced necrosis of tumor cells leading to the release of HMGB1, which is crucial for DC activation. This resulted in DC maturation and infiltration into draining LNs for the activation of tumor-specific T cells. Additionally, increased HMGB1 levels measured in sera of patients treated with IL PV-10 suggests that HMGB1 may be involved in eliciting a systemic immune response in patients. We have shown that circulating T cell populations and tumor-specific CD8^+^ T cells are increased in melanoma patients after IL PV-10 therapy. Together these results support the design of additional clinical studies to measure anti-tumor immune responses after IL injection of PV-10 in patients with melanoma.

## MATERIALS AND METHODS

### Animals

Female C57BL/6 mice (6–8 weeks old) were purchased from Harlan Laboratories. Mice were housed at the Animal Research Facility of the H. Lee Moffitt Cancer Center and Research Institute. Mice were humanely euthanized by CO_2_ inhalation according to the American Veterinary Medical Association Guidelines. Mice were observed daily and were humanely euthanized if a solitary subcutaneous tumor exceeded 200 mm^2^ in area or mice showed signs referable to metastatic cancer. All animal experiments were approved by the Institutional Animal Care and Use Committee and performed in accordance with the U.S. Public Health Service policy and National Research Council guidelines.

### Cell lines and cell culture

NIH3T3, 293T and melanoma B16 cells were obtained from American Type Culture Collection (ATCC). Human melanoma cells (526, 624 and 888) were obtained from the NIH. M05 tumor was generated by transfection of B16 melanoma with pAc-neo-OVA plasmid and was provided by Dr. Kenneth Rock (Dana-Farber Cancer Institute). M05 cells were maintained in media supplemented with 0.8 mg/ml G418 [36]. B16-F10-luc-G5 cells stably expressing firefly luciferase were obtained from Caliper Life Sciences. All cell lines were passaged less than 10 times after initial revival from frozen stocks and tested negative for mycoplasma contamination.

### Tumor supernatants and tumor digestion

PV-10 was provided by Provectus Biopharmaceuticals. Tumor cells were incubated with various doses of PV-10 for the indicated time *in vitro* and cell supernatant were collected. For generation of tumor explant supernatants (TES), when tumors reached 100-150 mm^2^, 50 ul of PBS or PV-10 were IL injected. After 24 hours, tumors were collected. Single-cell suspensions were obtained by passing cells through cell strainers and cultured at 2 million cells per ml for 24 hours. TES were collected after centrifugation.

For tumor digestion, tumors were isolated from tumor-bearing mice treated with PV-10 or PBS and were digested with tumor dissociation buffer (Miltenyi Biotec) and GentleMACS (Miltenyi Biotec). After lysis of RBCs, single-cell suspensions were analyzed by FACS.

### Flow cytometry, cell apoptosis assay and tetramer staining

Single-cell suspensions from the indicated tissues were prepared by pressing cells through a 70 μm cell strainer. After RBC lysis, cells were stained in FACS buffer with the following antibodies for flow cytometric analysis: anti-human CD3, CD4, CD8, CD25, CD11b, HLA-DR, CD14, FOXP3 and CD56; anti-mouse CD11c, I-A^b^, CD45.1, CD45.2, CD8, CD4, CD3, CD86, CD80 and CD40 (all from BD Bioscience). Cell apoptosis assays were performed by using Annexin V apoptosis detection kits (ebioscience). For tetramer staining, cells were stained with H-2K^b^ /SIINFEKL tetramer (MBL international) at room temperature for 20 minutes, followed by an additional 20 minute incubation with additional antibodies on ice. Live/dead fixable near-IR or aqua fluorescent reactive dyes (Thermo Fisher Scientific) were used to exclude dead cells before analysis. Cells were acquired by LSR II equipped with four lasers (BD Biosciences), and the data were analyzed with FlowJo (Tree Star).

### ELISA

For detection of IFN-γ in mouse samples, splenocytes from PBS- or PV-10-treated M05-bearing mice were expanded with 1 μg/ml SIINFEKL peptide, 20 ng/ml IL-15 and 20 ng/ml IL-21 (R&D Systems) and then mixed with irradiated M05 cells at a ratio of 10:1 [49]. For detection of IFN-γ from human samples, CD8^+^ T cells were isolated from peripheral blood mononuclear cells (PBMCs) with a human CD8^+^ T cell isolation kit (Miltenyi Biotec). Cells were co-cultured with tumor cells in triplicate at a ratio of 1:1. IFN-γ production in the supernatants was measured after 48 hours with an IFN-γ ELISA kit (BD bioscience). For detection of DAMPs in cell supernatants or in patient serum, an ELISA kit for HMGB1 (IBL international), IL-1α or HSP70 ELISA kit (both from R&D Systems) was used.

### Induction of lung and subcutaneous B16 lesions

C57BL/6 mice were injected i.v. with 5e5 viable B16-F10-luc-G5 and s.c. in the left flank with 1e5 B16-F10 cells. Seven days later, mice were treated by IL injection of the subcutaneous tumor with 50 μl PV-10 or PBS. Mice were treated with 100 μg of purified CD8 depleting antibody (2.43, BioXcell) or isotype (rat IgG_2b_) 2 and 3 days prior to PV-10 treatment and twice per week thereafter. Mice were shaved, intraperitoneally injected with luciferin (150 mg/kg of body weight) and imaged with Xenogen IVIS® 200 Image Series (PerkinElmer) on day 21.

### Adoptive transfer of T cells

CD45.1^+^ OT-1 T cells were purified with a T cell enrichment column (CD3^+^ T cell purity >90%) (R&D Systems) and were incubated with CellTracer™ Violet (Thermo Fisher Scientific) for 20 min at 37°C. After two washes in PBS, 3e5 labeled cells were resuspended in 100 μl of PBS and injected i.v. into MO5 tumor-bearing mice. After 4 days, spleens, lymph nodes (LNs) and tumors were harvested and stained with CD45.1, CD45.2 and CD3 antibodies. Cells were gated on CD45.1^+^CD45.2^−^CD3^+^ and cells with at least one division were considered “divided cells”.

### Dendritic cell enrichment

M05 melanoma tumor-bearing mice were treated with IL PV-10 or PBS on day 7. After 18 hours, mice were sacrificed and tumor draining LNs were dissected and pooled for single cell preparation. DCs were enriched from pooled samples with a Pan Dendritic cell isolation kit (Miltenyi Biotec) and stained with antibodies against CD11c, MHC II, CD40, CD86 and CD80. Cells were analyzed on live CD11c^+^ MHC II^+^ cells.

### T cell proliferation assay

Bone marrow derived DCs were purified by Opti-prep gradient (Axis-Shield) after 5 days of BM culture with 10 ng/ml GM-CSF and 20 ng/ml GM-CSF and 10 ng/ml IL-4 [50, 51]. DCs were cultured for 2 days with GM-CSF, IL-4 and 20% tumor supernatants (as described above). Next, DCs were pulsed for 2 hours with 10 μg/ml of OVA protein (Sigma-Aldrich). After multiple washes, DCs were co-cultured with 1e5 responder OT-1 T cells in triplicate, in U-bottom 96-well plates at different stimulator-to-responder ratios for 3 days. ^3^H-thymidine (1 μCi) was added to each well 18 hours prior to cell harvesting. T cell proliferation was measured by ^3^H-thymidine incorporation in a liquid scintillation counter Microbeta® Trilux (PerkinElmer).

### HMGB-1 blockade

BM-derived DCs and 20% tumor supernatants were separately incubated with an antagonistic antibody against HMGB1 (IBL international) or the relevant isotype for 4 hours, and then were mixed together for culture for 2 days. After multiple washes, DCs were pulsed with OVA protein and co-cultured with OT-1 T cells for T cell proliferation as described above.

### Determination of IC50

Cells were incubated in the dark with 12.5, 25, 50, 100, or 200 μM PV-10 or PBS in a 12-well plate for 6, 12, 24 and 48 hours. All cells in wells were collected, mixed with counting beads and acquired by LSR II. DAPI was used to exclude dead cells before analysis. The absolute number of live cells was calculated by comparing the ratio of bead events to cell events. The half maximal inhibition of PV-10 on cell growth was determined as IC50 using GraphPad Prism.

### Human subjects

Fifteen patients with dermal and/or subcutaneous metastatic melanoma were enrolled in a pilot study (NCT01760499). Peripheral blood and serum were collected prior to biopsy, 7-14 days after IL PV-10 injection into a single melamona tumor, and 21-28 days after IL PV-10 injection. PBMCs were isolated by Ficoll–Paque Plus (GE healthcare). Blood samples were sent for HLA typing to determinate HLA-matched tumor and HLA mismatched tumor for each patient. Serum was prepared by collecting the supernatant after incubation of blood at room temperature for 1 hour and centrifugation at 1,000 g. Two tumor lesions in each patient were sampled by biopsy pre-treatment; one of the two lesions was injected with IL PV-10 7 days after biopsy, then both residual sites were completely excised 7-14 days later. Biopsy specimens were fixed in formalin and embed in paraffin. The specimens were stained with hematoxylin and eosin stains for determination of pathologic complete response. Immunohistochemistry for melanin A (mel A) was performed. Flow cytometry was performed to detect CD3, CD4, CD8, and CD56 staining on PBMC.

### Statistical analysis

The data were analyzed with a two-tailed Student *t*-test or Wilcoxon matched pairs test by GraphPad Prism. A *p* value of < 0.05 was considered statistically significant.

## SUPPLEMENTARY FIGURES


