# Supplementary material for: Intralesional rose bengal in melanoma elicits tumor immunity via activation of dendritic cells by the release of high mobility group box 1
Source: Oncotarget. 2016 May 9;7(25):37893–905. doi: 10.18632/oncotarget.9247 (PMC5122358; doi:10.18632/oncotarget.9247)
Supplement: Supplementary file 1 [file oncotarget-07-37893-s001.pdf]

SUPPLEMENTARY FIGURES

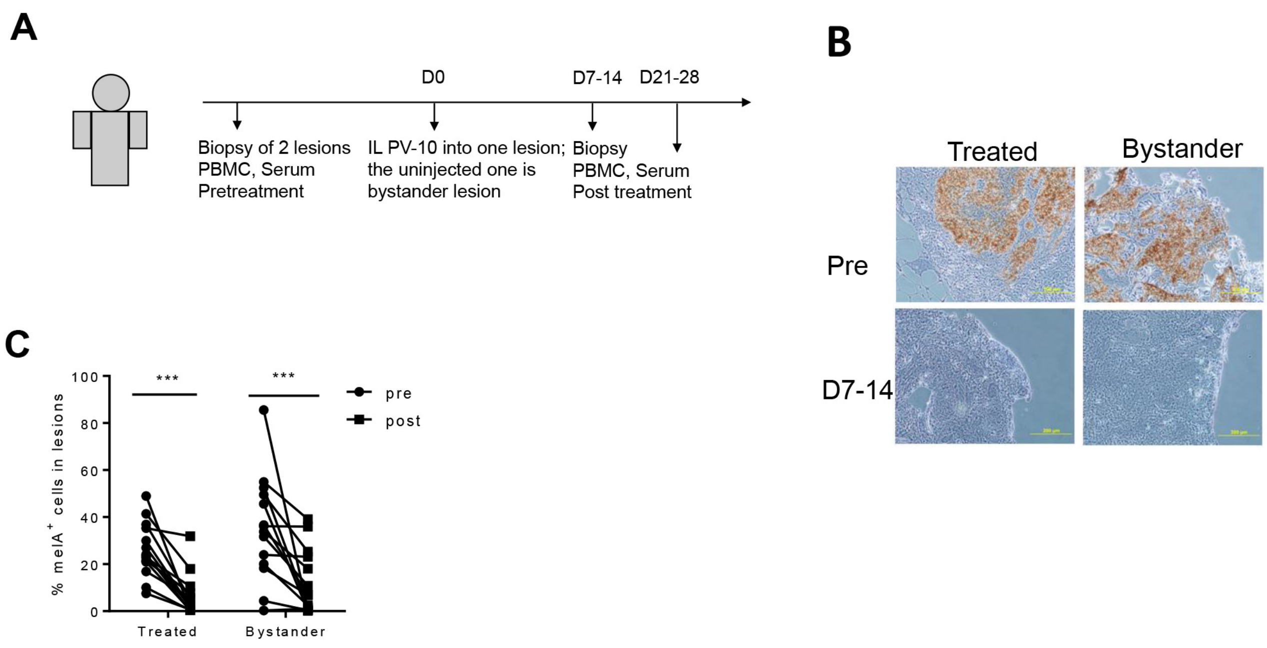

Supplementary Figure S1: A systemic immune response in melanoma patients treated with IL PV-10. A. Clinical study design. **B, C.** Biopsies of treated and bystander lesions were stained with antibodies against melA to determine the presence of melanoma cells (n=14 patients). Data are shown as mean ± SEM. *P* values were determined by Wilcoxon matched-pairs signed rank test. ***, $p<0.001$. The presence of tumor cells in the treated and bystander lesion was confirmed by a pathologist.

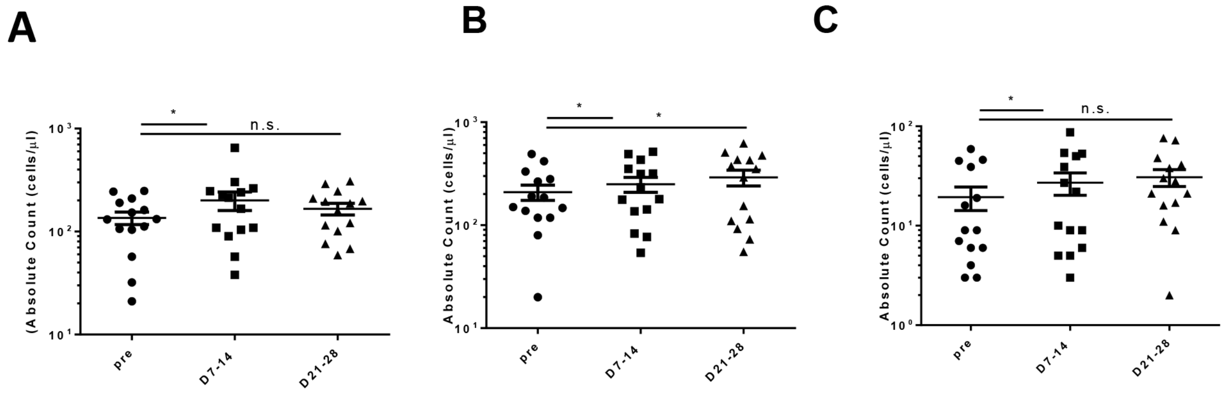

Supplementary Figure S2: Increased circulating T and NKT cells after IL PV-10 treatments. A. CD8+ T cells, **B.** CD4+ T cells, and **C.** NKT cells in PBMCs of patients increased after IL PV-10 treatment (n=14 patients). Data show the absolute numbers of live cells per ml of peripheral blood. *P* values were determined by Wilcoxon matched-pairs signed rank test. *, $p<0.05$, statistically significant versus pre-treatment; n.s., not significant.

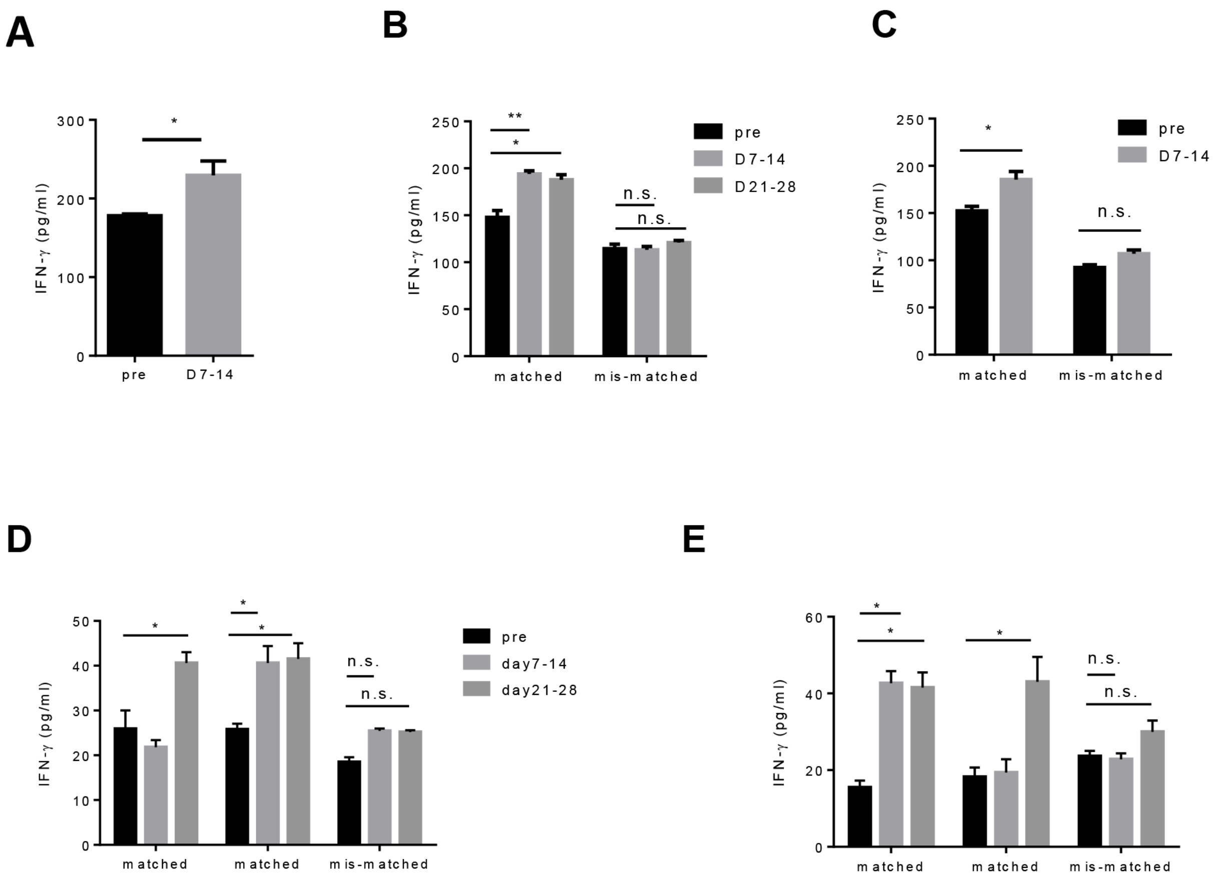

Supplementary Figure S3: Increased tumor-specific T cell response after IL PV-10 treatment. IFN-γ production from CD8+ T cells purified from PBMCs from 5 patients and re-stimulated with autologous melanoma cells **A.** or HLA- matched or HLA-mismatched melanoma cells **B-E.** Data were measured in 3 independent experiments with triplicates for each experiment. *P* values were determined by an unpaired student *t*-test. *, p<0.05 statistically significant versus pre-treatment; **, p<0.01; n.s., not significant.
